# Sex-Specific Patterns of Aberrant Brain Function in First-Episode Treatment-Naive Patients with Schizophrenia

**DOI:** 10.3390/ijms160716125

**Published:** 2015-07-16

**Authors:** Wei Lei, Mingli Li, Wei Deng, Yi Zhou, Xiaohong Ma, Qiang Wang, Wanjun Guo, Yinfei Li, Lijun Jiang, Yuanyuan Han, Chaohua Huang, Xun Hu, Tao Li

**Affiliations:** 1 The Mental Health Center & Psychiatric Laboratory, State Key Laboratory of Biotherapy, West China Hospital, Sichuan University, Chengdu 610041, China; E-Mails: lw17022@163.com (W.L.); xinzhishui@tom.com (M.L.); mrdengwei@163.com (W.D.) maxiaohong@scu.edu.cn (X.M.); wangqiang130@hotmail.com (Q.W.); guowjcn@163.com (W.G.) li-yinfei@163.com (Y.L.); doctorjiangli@163.com (L.J.); maggiehyy2011@hotmail (Y.H.); chaohua118@gmail.com (C.H.); 2 Hospital of Chengdu Office of People’s Government of Tibetan Autonomous Region, Branch Hospital of West China Hospital, Sichuan University, Chengdu 610041, China; E-Mail: znxc119@sina.com; 3 Biobank, West China Hospital, Sichuan University, Chengdu 610041, China; E-Mail: hxxhu99@gmail.com

**Keywords:** schizophrenia, functional MRI, sex difference, brain functional network

## Abstract

Male and female patients with schizophrenia show significant differences in a number of important clinical features, yet the neural substrates of these differences are still poorly understood. Here we explored the sex differences in the brain functional aberrations in 124 treatment-naïve patients with first-episode schizophrenia (61 males), compared with 102 age-matched healthy controls (50 males). Maps of degree centrality (*DC*) and amplitude of low-frequency fluctuations (*ALFF*) were constructed using resting-state functional magnetic resonance imaging data and compared between groups. We found that: (1) Selective *DC* reduction was observed in the right putamen (Put_R) in male patients and the left middle frontal gyrus (MFG) in female patients; (2) Functional connectivity analysis (using Put_R and MFG as seeds) found that male and female patients have disturbed functional integration in two separate networks, *i.e.*, the sensorimotor network and the default mode network; (3) Significant *ALFF* alterations were also observed in these two networks in both genders; (4) Sex specific brain functional alterations were associated with various symptoms in patients. These results suggested that sex-specific patterns of functional aberration existed in schizophrenia, and these patterns were associated with the clinical features both in male and female patients.

## 1. Introduction

Male and female patients with schizophrenia show significant differences in a number of important clinical features, including premorbid functioning, age of onset, clinical presentation, illness course and response to treatment [1,2]. Compared with male patients, females with schizophrenia typically show better premorbid adjustment [3], a less deteriorative course of illness [4,5], and better treatment response [6] and outcomes [7]. In addition, some studies have identified that male patients scoring higher in both quantity and severity of negative symptoms [8,9], and female patients experiencing more severe affective symptoms, such as anxiety and depression [9,10].

There has been great interest in exploring neural mechanisms underpinning the sex-related differences in schizophrenia. In general, previous investigations have found greater structural brain abnormalities in male patients. Compared to females, male patients tend to have larger lateral and third ventricles, smaller volumes of the frontal and temporal lobe, and greater left-lateralized abnormalities [11,12]. Several studies have explored sex differences in the alterations of brain function in schizophrenia using task-based functional magnetic resonance imaging (fMRI) and electroencephalography (EEG). These studies have revealed sexual dimorphism in the disturbances of brain activity in patients with schizophrenia, involving memory [13], mental rotation [14,15] and emotional processing [16].

However, it is difficult to reach generalizable conclusions from task-based fMRI research, due to the variable processing involved in different tasks [17]. Unlike the task-based approach, resting-state fMRI (rfMRI) provides assessments of spontaneous activity of the brain in subjects not performing a task. rfMRI avoids not only the above confounds of task and processing differences, but is also relatively easy to carry out, and thus warrants further clinical application. In addition, low frequency (0.01–0.08 Hz) fluctuations (LFF) of the blood oxygenation level-dependent (BOLD) signal in rfMRI are considered to be physiologically meaningful and related to intrinsic neural activity [18].

There has been a dramatic increase in rfMRI-based schizophrenia studies during the last decade. It has been reported that both inter-regional synchronization (referred to as functional connectivity) and the amplitude of LFF (*ALFF*) are disrupted in schizophrenia [19,20]. Studies of functional connectivity by rfMRI have revealed that the brain functional networks in patients with schizophrenia are associated with a less hub-dominated, and less integrated configuration, compared to healthy controls [21,22]. The disturbed integrity of functional networks is associated with impaired higher-order cognitive functions and psychotic symptoms [23] in patients with schizophrenia. Degree centrality (*DC*) analysis is a voxel-wise, data-driven method that allows the mapping of functional integration in the brain at the voxel level. This measure has been widely used to examine the altered integration of networks in functional diseases of the brain [24,25]. The reproducibility of *DC* analysis has been demonstrated for different test durations and in both passive and active task states [25,26]. However, no previous study has investigated the sexual dimorphisms in the disturbances of functional network integration in schizophrenia.

Previous studies have suggested that the amplitude of the BOLD response is significantly correlated with the local field potential activity [27], and that the *ALFF* was related to regional spontaneous neuronal activity [28]. Alterations in the *ALFF* have been reported frequently in patients with schizophrenia [29,30,31], indicating a disruption in the intrinsic brain activity. *ALFF* measurements have also shown moderate-to-high test-retest reliability when assessed twice, over a median interval of 2.5 months, in patients with chronic schizophrenia [32].

The purpose of the present study was to explore the sex differences in the functional aberrations of the brain in patients with first-episode schizophrenia (FES), using *DC* and *ALFF* measures of rfMRI. The clinical symptoms of patients were evaluated using the Positive and Negative Syndrome Scale (PANSS) [33], which provided the total score and five syndrome scores including negative, positive, excited, depressed, and disorganized/concrete syndrome [34]. Majority of FES patients were treatment-naive to eliminate potential effects of antipsychotics in current study. Based on the existing literature, we hypothesized that: (1) males and females with FES may exhibit different patterns of brain functional aberration, albeit with a fair amount of overlap; and (2) the sexually dimorphic patterns of brain functional aberration are associated with the clinical features.

## 2. Results and Discussion

### 2.1. Demographic, Clinical and Head-Motion Characteristics

There were no significant differences between any of the groups in age and head motion parameters. Furthermore, there were no significant differences between male and female patients with FES in the age at onset, duration of untreated psychosis and PNASS scores (Table 1).

### 2.2. DC

**Figure 1:**
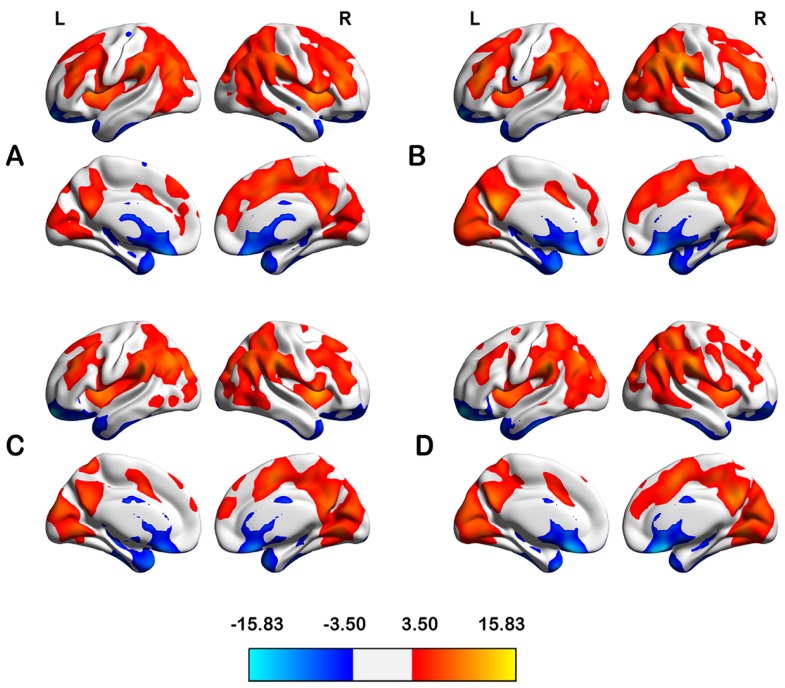
Degree centrality (*DC*) maps within groups of male HC (HCm) (**A**); male FES (FESm) (**B**); female HC (HCf) (**C**) and female FES (FESf) (**D**), the color bars represent the *t*-values of one sample *t*-test in *DC* maps. FES, first episode schizophrenia patients; HC, healthy controls; L, left hemisphere; R, right left hemisphere.

In HCs, the highly connected regions (considered as candidate hubs) were located primarily bilaterally in the dorsolateral prefrontal cortex, inferior parietal lobule, superior and medial temporal gyri, precuneus, posterior cingulate cortex, insula and striatum. Notably, the spatial distributions of the candidate hubs were similar across all four groups, despite some differences in strength (Figure 1).

**Figure 2:**
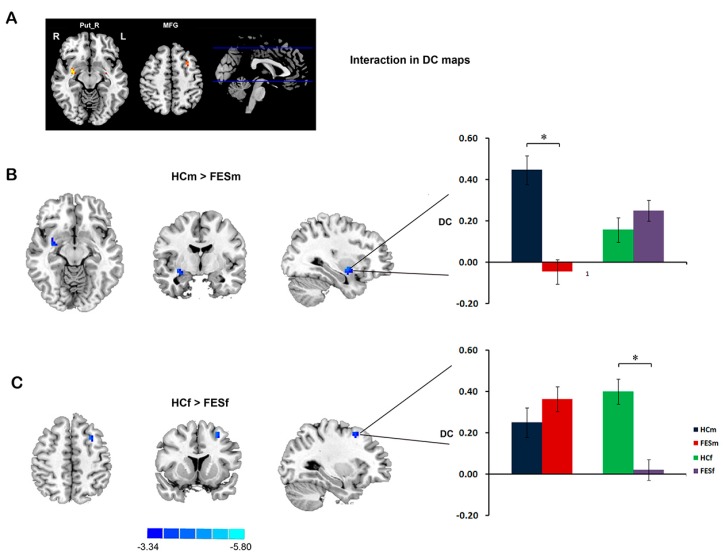
Sex by diagnosis interaction in *DC* maps. Significant sex by diagnosis interaction was found in right putamen (Put_R) and left middle frontal gyrus (MFG) (**A**); Simple effect analysis suggested that male FES patients showing selective *DC* reduction in Put_R (**B**); while female patients showing selective *DC* reduction in MFG (**C**). The color bars represent the *t*-values in *post hoc* comparisons. The bar chart indicates the average *z*-transformed *DC* of ROIs (Region of interest) for each group. * Represent a significant difference detected. L, left hemisphere; R, right left hemisphere.

The regions showing significant differences between groups in the *DC* are listed in Table 2. Compared with controls, patients with FES showed decreased *DC* in the right putamen (Put_R) and inferior frontal gyrus. Significant sex by diagnosis interactions were found in the Put_R and left middle frontal gyrus (MFG). *Post hoc* pair-wise comparisons showed that the significant *DC* reduction in the Put_R occurred selectively in male patients, while the *DC* reduction in the MFG occurred selectively in female patients (Figure 2). According to the main effect of sex, males showed a higher *DC* in the superior and inferior frontal gyri and PCC, but a lower *DC* in the IPL, thalamus and insula, than females.

**Table 1:** Demographic and clinical characteristics of participants.

Items	FESm (*n* = 61)	FESf (*n* = 63)	HCm (*n* = 50)	HCf (*n* = 52)	*p*
Age	24.31 (6.57)	24.62 (6.82)	24.80 (6.74)	24.71 (6.98)	0.982
Education years	13.13 (2.49)	12.94 (2.49)	13.77 (2.91)	13.40 (2.55)	0.366
HM-tran (mm)	0.13 (0.14)	0.11 (0.13)	0.11 (0.11)	0.10 (0.10)	0.099
HM-rota (degree)	0.12 (0.12)	0.11 (0.14)	0.11 (0.09)	0.11 (0.11)	0.649
Total	87.59 (17.07)	89.27 (17.04)	–	–	0.597
Positive	14.74 (4.40)	15.77 (4.24)	–	–	0.200
Negative	16.58 (7.73)	14.85 (8.39)	–	–	0.250
D/C	9.12 (2.75)	9.48 (2.96)	–	–	0.497
Excited	9.93 (3.85)	11.32 (4.13)	–	–	0.063
Depressed	6.19 (3.08)	6.12 (2.71)	–	–	0.887
DUP (months)	8.19 (8.97)	5.50 (8.03)	–	–	0.118
Age of Onset	23.24 (6.65)	23.76 (6.64)	–	–	0.689

Demographic data are shown as mean (standard deviation). FES, first episode schizophrenia patients; HC, healthy controls; FESm, male FES; FESf, female FES; HCm, male HC; HCf, female HC; HM-tran, transnational head-motion; HM-rota, rotational head-motion; Total, the Positive and Negative Syndrome Scale (PANSS) total scores; Positive, PANSS positive syndrome scores; Negative, PANSS negative syndrome scores; D/C, PANSS disorganized/concrete syndrome scores; Excited, PANSS excited syndrome scores; Depressed, PANSS depressed syndrome scores; DUP, duration of untreated psychosis.

**Table 2:** Sex and diagnosis effects on voxel-wise *DC* characteristics.

Comparisons	Regions	Voxels	*t* Value *	*X*, *Y*, *Z*
Interaction of sex and diagnosis				
HCm > FESm	Putamen	22	5.24	30, −3, −12
HCf > FESf	Middle Frontal Gyrus	13	4.40	−30, 12, 51
Main effect of diagnosis				
HC > FES	Putamen	63	4.60	36, −15, −6
	Inferior Frontal Gyrus	35	3.84	36, 3, 30
Main effect of sex				
Male > Female	Inferior Frontal Gyrus	62	4.83	−36, 36, 0
	Superior Frontal Gyrus	25	4.29	27, 33, 36
	Superior Frontal Gyrus	35	3.89	−21, 57, −15
	Posterior Cingulate Cortex	16	3.84	−6, −48, 24
Female > Male	Inferior Parietal Lobule	26	−3.34	−48, −36, 21
	Thalamus	99	−3.35	−9, −33, −3
	Thalamus	65	−3.38	9, −21, −6
	Insula	24	−3.41	−39, −3, −6

All clusters were identified use the threshold of *p* < 0.05 AlphaSim corrected (*i.e.*, *p* < 0.001 combined with a minimal cluster size of 13 voxels). * All clusters were identified using *post hoc* two-sample *t*-tests within a mask of *F*-contrast (interaction or main effects).

### 2.3. Networks

**Figure 3:**
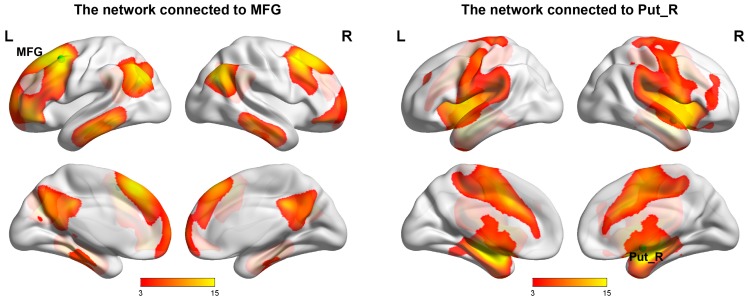
Functional networks that linked to MFG (**left panel**) and Put_R (**right panel**) in healthy controls. The seed regions showed as green dots. The color bars represent the *t*-values of voxel-wise one-sample *t*-tests. L, left hemisphere; R, right left hemisphere; Put_R, right putamen; MFG, middle frontal gyrus.

The Put_R and MFG were selected as seeds for network analysis. Figure 3 illustrates the networks linked to each of the seeds in healthy controls. Interestingly, the MFG and Put_R were components of two separated networks. Similar networks could be reconstructed in all four groups of this study, despite some differences in strength (Please see Figures S5 and S6).

The MFG-related network was similar to the previously described default mode network (DMN) [35]; brain regions in this network included IPL, PCC, medial prefrontal cortex (MPFC) (including the MFG cluster) and inferior temporal gyrus. The Put_R-related network was similar to the previously reported sensorimotor network (SMN) [36,37]; which included the primary and supplementary motor cortices, sensorimotor cortices, middle cingulate gyrus, striatum (including the Put_R cluster), middle-posterior insula and cerebellum (Please see also Figure S7 for superimposed maps of the “MFG-related network” and “Put_R-related network” with canonical SMN and DMN).

### 2.4. ALFF

**Figure 4:**
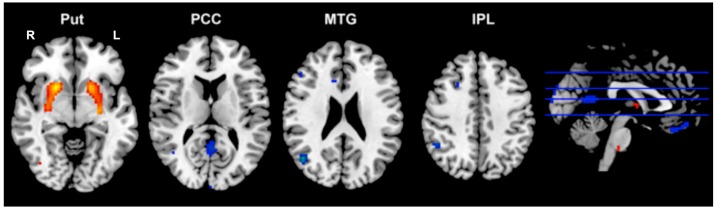
The amplitude of low-frequency fluctuations (*ALFF*) alterations in FES patients. The regions showing significant *ALFF* difference between patients with FES and HCs. L, left hemisphere; R, right left hemisphere.

The regions showing significant differences between groups in the *ALFF* are listed in Table 3. Compared with healthy controls, patients with FES showed significantly increased *ALFF* in the bilateral putamen, and significantly decreased *ALFF* in the posterior cingulate cortex (PCC), right middle temporal gyrus (MTG), right inferior parietal lobe (IPL) and ventromedial prefrontal cortex (vmPFC) (Figure 4).

**Table 3:** Sex and diagnosis effects on voxel-wise *ALFF* characteristics.

Comparisons	Regions	Voxels	*t* Value *	*X*, *Y*, *Z*
Interaction of sex and diagnosis				
HCf > FESf	Ventral Medial Prefrontal Cortex	58	4.51	−6, 30, −24
Main effect of diagnosis				
HC < FES	Putamen	315	5.81	21, 15, −6
	Putamen	363	5.37	−18, 15, −3
HC > FES	Middle Temporal Gyrus	52	4.84	45, −63, 24
	Inferior Parietal Lobule	27	4.06	48, −48, 42
	Posterior Cingulate Cortex	38	3.99	0, −54, 12
	Ventral Medial Prefrontal Cortex	34	3.61	−3, 40, −21
Main effect of sex				
Male > Female	Inferior Parietal Lobule	3709	9.61	54, −51, 48
	Middle Frontal Gyrus	257	6.06	−45, 48, 18
	Cerebellar Tonsil	56	5.24	12, −42, −51
	Middle Frontal Gyrus	108	5.21	48, 48, 15
	Lingual Gyrus	51	4.02	−15, −102, −9
	Superior Temporal Gyrus	53	5.20	57, 15, −6
	Insula	51	4.70	−39, −33, 12
Female > Male	ParaHippocampal	105	5.08	18, −12, −36
	Hippocampus	129	4.78	33, −33, −9
	Lentiform Nucleus	731	6.41	−15, 9, −3
	Cerebellum Posterior Lobe	2966	6.49	39, −39, −45

All clusters were identified use the threshold of *p <* 0.05 AlphaSim corrected (*i.e.*, *p <* 0.001 combined with a minimal cluster size of 13 voxels). * All clusters were identified using two-sample *t*-tests within masks from *F*-contrasts (interaction or main effects).

**Figure 5:**
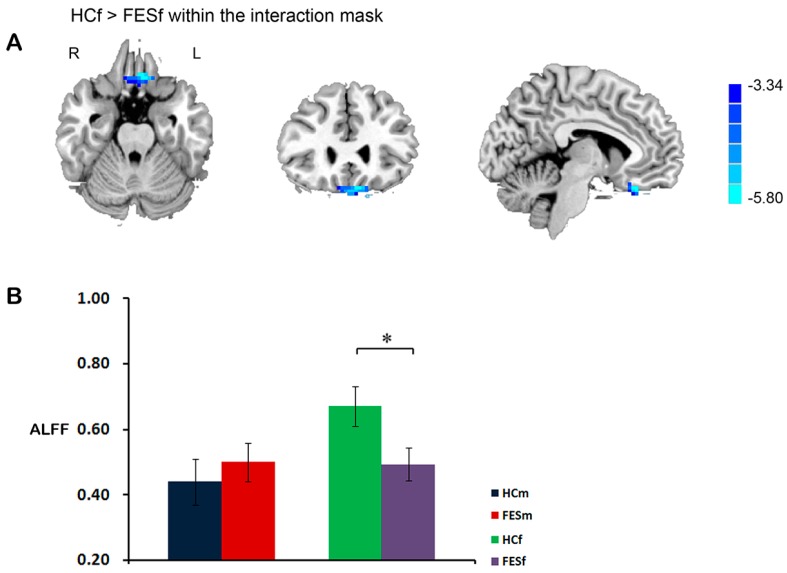
Female FES patients showing selective *ALFF* reduction in ventral medial frontal gyrus (vmPFC). The cluster was identified using two-sample *t*-test, HCf *vs.* FESf, within a mask that showing significant sex by diagnosis interaction (**A**); The bar chart indicates the average *z*-transformed *ALFF* of vmPFC for each group (**B**). * Represents a significant difference was detected.

Significant sex by diagnosis interaction was found in the vmPFC. *Post hoc* comparisons within this mask revealed that female patients, but not male patients, showed a reduction in the *ALFF* in this region, compared with healthy controls of the same sex (Figure 5).

According to the main effect of sex, males exhibited higher *ALFF* values predominantly in the parietal and frontal regions, and lower *ALFF* values in the lentiform nucleus and cerebellar posterior lobe, compared with females.

### 2.5. Correlations of Regional Functional Characteristic with Clinical Profiles

The mean *DC* values of the Put_R and MFG; and the mean *ALFF* values of the left and right putamen, vmPFC, PCC, MTG and IPL clusters were extracted and correlated with clinical measures. We found that, *DC* value in Put_R was positively correlated with syndromes of depressed (*r* = 0.379, *p* = 0.004) in male patients. In combining patient group (with both male and female patients), *DC* value in MFG was negatively correlated with syndromes of excited (*r* = 0.258, *p* = 0.005); *ALFF* in the vmPFC was correlated with syndromes of depressed (*r* = 0.269, *p* = 0.003); *ALFF* in the bilateral putamen of patients with FES correlated with syndromes of disorganized/concrete (*r* = 0.318, *p* = 0.000 and *r* = 0.297, *p* = 0.001 for the left and right putamen, respectively) and marginally correlated with negative symptoms (*r* = 0.226, *p* = 0.014 and *r* = 0.234, *p* = 0.011 for the left and right putamen, respectively). No other significant correlations were detected.

## 3. Discussion

The present study found that: (1) Selective *DC* reduction was observed in the right putamen (Put_R) in male patients and the left middle frontal gyrus (MFG) in female patients; (2) Functional connectivity analysis (using Put_R and MFG as seeds) found that male and female patients have disturbed functional integration in two separate networks, *i.e.*, the sensorimotor network and the default mode network; (3) Significant *ALFF* alterations were also observed in these two networks in both genders; (4) Sex specific brain functional alterations were associated with various of symptoms of patients.

The most significant advantage of the present study is the recruitment of a relatively large group of patients with FES, and the majority of them are treatment-naive. Significant effects of antipsychotics on the striatal areas such as the putamen and the MPFC have been reported [38]. The present study thus allowed eliminating potential effects of antipsychotics. For part of patients who have been minimally treated before scan, re-analysis with these patients excluded did not qualitatively change our results.

The dysconnectivity hypothesis suggests that the core pathology of schizophrenia is related to disrupted functional integrity between distinct brain regions [39]. In line with this concept, the present study identified a significant reduction in the integrity of the network related to right putamen and left MFG in patients with FES. Moreover, the pattern of disrupted functional integrity showed interactions with the gender of the patient: selective *DC* reduction was observed in the right putamen of male patients and the left MFG of female patients. Functional connectivity analysis of these two regions of interest suggested that they were the hubs of two separated networks, *i.e.*, the putamen was the hub of the SMN while the MFG was the hub of the DMN. These results are consistent with findings from previous studies. A reduction in the functional connectivity strength (a measure that is similar to the *DC*) in the SMN, an increase in the diversity in the striatum [21], and an aberrant functional connectivity in the DMN [40] have all been reported to occurred in patients with schizophrenia. Taken together, our results suggest a sex-specific pattern of integrity disruption in two networks, namely the SMN in males and the DMN in females.

Consistently, our *ALFF* analysis showed that the alterations in regional intrinsic activity in patients with FES mainly involved regions of the DMN (vmPFC, PCC and IPL) and SMN (bilateral putamen). Several rfMRI studies have investigated disturbed intrinsic neural activity in schizophrenia. Increased intrinsic neural activity in the putamen has been consistently reported in both drug-naive patients with FES [29,30,41,42] and patients with chronic schizophrenia [43]. *ALFF* reductions in patients with FES have been shown to be highly repeatable observations in the vmPFC [29,30,31,41], although less so in the IPL [29,30] and PCC. Our results are in line with those of previous reports suggesting that aberrant intrinsic brain function in the DMN and SMN may represent an important aspect of patients with FES.

The putamen is involved in dopamine metabolism, and has been implicated as a core component in the pathophysiology of schizophrenia [44]. The increases in striatal dopamine synthetic capacity [45] and striatal dopamine transmission [46] in drug-naive patients with FES suggest that a hyperdopaminergic state in the striatum at illness onset may represent a crucial aspect of schizophrenia. It has been postulated that the hyperdopaminergic state in the striatum, which is related with dopaminergic depletion in the cortex, might represent the biochemical basis for negative symptoms in schizophrenia [47]. In line with this hypothesis, a worsening of negative symptoms has been associated with an increased availability of striatal dopamine D2 receptors in longitudinal studies [48]. In fact, functional aberration of the whole SMN has been suggested to be the common pathology underlying negative symptoms in major depression, schizophrenia and Parkinson’s disease [49]. In the present study, the intrinsic activity of the putamen was (marginally) associated with negative symptoms in patients with FES. These results suggest that the SMN (in which male patients showed a dominant integration disturbance) is associated with negative symptoms in schizophrenia. This notion is also consistent with previous findings that male patients tend to show more severe negative symptoms than females [8,9].

Our results revealed that female patients dominated integrity disruption in the DMN, and also showed a selective *ALFF* reduction in the vmPFC. The DMN is involved in multiple social emotional processes, such as social decision-making, emotional control and theory of mind [35], functional alterations in the DMN have also been related to the pathology of major depression, bipolar disorder and schizophrenia [40,41].

In particular, by receiving inputs from the temporal association cortex, amygdala and hypothalamus, the vmPFC is one of the highest integrating centers for emotional reasoning and participates in almost all theory of mind tasks [50]. The functional abnormalities in vmPFC have also been associated with impaired theory of mind in schizophrenia [51]. The MFG is believed to be involved in processing cognitive aspects of theory of mind [52]. Likewise, functional abnormalities in the IPL have also been consistently associated with the impairments in emotional perception and theory of mind reported in schizophrenia [53]. In fact, the vmPFC, MFG, PCC and IPL have all been suggested to be part of the neural network for processing theory of mind tasks [54]. Consistent with our results, impaired theory of mind has been frequently reported in patients with schizophrenia [55]. The female-dominated functional aberration in the DMN thus may be associated with the more severe emotional symptoms in female patients [9,10]. Indeed a recent meta-analysis suggests that emotion processing impairments are significant moderated by sex, with male patients showed more impairment in emotion identification and stronger emotion identification-outcome association [56]. The correlations of *ALFF* in vmPFC with syndromes of depressed, and *DC* in MFG with excited found in present study also support the association of functional changes with emotion processes in patients. Female specific functional alterations of these regions may underpin previous reported sex differences in the event-related potential (ERP) correlations of depression and excitement symptoms in schizophrenia [57,58].

Several methodological issues should be considered when interpreting these results. The first limitation of the present study was the recruitment of in-patients. Given that the in-patient population is commonly more ill than out-patients, this approach may thus have led to a bias in the selection of female patients with advanced disease rather than “average” females with schizophrenia [1]. This approach may limit the generalizability of our findings to general population with schizophrenia. Second, the recruitment of patients with FES provided an advantage in terms of controlling potential confounders, such as the effects of medication and long-term illness course. However, this approach may also have resulted in our sample of patients being atypical from general clinical patients with schizophrenia. Patients that were too unwell to participate in the study (e.g., patients with very severe positive symptoms or aggression) were excluded, which may have led to bias toward the selection of patients with an atypical illness condition. However, there is no practical way to study the long-term course of schizophrenia in un-medicated patients, hence we consider the study of patients with FES to be the best option.

## 4. Experimental Section

### 4.1. Participants

A total of 145 treatment-naive patients with FES and 116 age- and sex-matched healthy control (HC) subjects participated in the present study. The subjects were recruited, between 2008 and 2013, at the Mental Health Centre of the West China Hospital, Sichuan University (Chengdu, China). The patients were assessed by qualified psychiatrists using the Structured Clinical Interview for DSM-IV (Patient Edition) (SCID-I/P) [59] shortly after presentation to the mental health services. Diagnoses were assigned according to the diagnostic criteria for schizophrenia as specified in DSM-IV. All patients also underwent further evaluation of their clinical symptoms using the Positive and Negative Syndrome Scale (PANSS) [33], which provided the total score and five syndrome scores including Negative, Positive, Excited, Depressed, and Disorganized/concrete factor [34].

HCs were recruited from the local area by poster advertisement. All HCs were screened for a lifetime absence of major psychiatric illnesses by using the SCID non-patient version (SCID-I/NP) [60]. In addition, the HCs were interviewed to ascertain that there was no psychiatric illness in their first-degree relatives.

The following exclusion criteria applied to all subjects: head trauma; severe physical diseases; pregnancy; current substance abuse; intellectual disability; neurological disorders; or excessive head movement (translational movement > 1.5 mm or rotation > 1.5°) during rfMRI scanning. Thirty-five subjects (21 patients and 14 controls) were excluded due to excessive head movement during rfMRI. The remaining 124 patients with FES and 102 healthy controls were assigned into 4 subgroups according to their sex, *i.e.*, male patients with FES (FESm, *n* = 61), female patients with FES (FESf, *n* = 63), male HCs (HCm, *n* = 50) and female HCs (HCf, *n* = 52). Twenty-eight of the included patients (22.58%; 15 male and 13 female) had been minimally treated with low dose antipsychotics (risperidone or olanzapine; 25 to 75 mg of chlorpromazine daily dose equivalent) for less than 3 days prior to MRI scanning, the remaining patients (77.42%) were neuroleptic-naive before scanning. Re-analysis with these 28 patients excluded did not qualitatively change our results (Figure S1). All participants were Han Chinese and right-handed, according to Annette’s handedness test [61]. This study was approved by the Institutional Review Board of West China Hospital, Sichuan University (National Nature Science Foundation of China projects No. 30530300 and 81130024, started on 1 January 2005 and 1 January 2011 respectively). All subjects gave their informed consent before participating in the study.

### 4.2. MRI Data Acquisition

Subjects were scanned using an Excite 3T MRI system (General Electric, Milwaukee, WI, USA) with an 8-channel phased-array head coil. rfMRI images were obtained using an echo-planar imaging sequence (repetition time (*TR*) = 2000 ms; echo time (*TE*) = 30 ms; flip angle = 90°; slice thickness = 5 mm (no gap); field of view (*FOV*) = 240 × 240 mm^2^; matrix = 64 × 64). Each brain volume comprised 30 axial slices, and each functional run contained 200 image volumes. During scanning, participants were instructed to lie still with their eyes closed, remain relaxed, and refrain from focusing on any particular thought.

To assist preprocessing of rfMRI images, high-resolution T1-weighted images were also obtained with a spoiled gradient recall sequence (*TR* = 8.5 ms; *TE* = 3.4 ms; flip angle = 12°; slice thickness = 1 mm (no gap); *FOV* = 240 × 240 mm^2^; matrix = 256 × 128) producing 156 contiguous axial slices cover the whole brain.

### 4.3. Image Preprocessing

The preprocessing was carried out using the DPARSF version 2.2 [62]. Specifically, the preprocessing analysis comprised: (1) discarding the first 5 volumes from each resting-state scan to allow for signal equilibration; (2) slice-dependent time shifts; (3) 3D motion correction; (4) nuisance signal regression (including the global mean, white matter, CSF signals, and 6 motion parameters: the global mean intensity, white matter and CSF signals were extracted using a mask, in native space, derived from uniform segmented T1 images of each subject); (5) linear trend removal; and (6) band-pass temporal filtering (0.01–0.08 Hz). The output of these preprocessing steps was a 4D residual functional volume in native functional space for each participant. These data were then spatially normalized to the MNI space and resampled at 3 mm cubic voxels.

### 4.4. DC Calculation

The *DC* was calculated using REST software [63]. Briefly, (1) the Pearson’s correlations between the time series of all pairs of brain voxels were calculated to obtain a whole-brain functional connectivity matrix for each participant. The computation was constrained within a gray-matter mask constructed from all subjects in the present study; (2) The functional connectivity matrix was then thresholded by a pre-selected threshold of Pearson’s *r* > 0.25 to obtain an undirected binarized matrix, whose element was 1 if the correlation between the two voxels was >0.25 and 0 otherwise. This threshold was chosen to avoid counting voxels that had low temporal correlation, and thus was attributable to signal noise. Different threshold selections did not qualitatively change the results of the present study (see Figure S2 for results from *r* thresholded at 0.00–0.30, with an interval of 0.05). Notably, the removal of global signal causes a shift in the distribution of the correlation coefficients and makes interpretation of the negative connectivity ambiguous [64], we thus restricted our explorations to positive correlations, as in previous studies [25]; (3) From the adjacency matrix, a map of the degree was computed by counting for each voxel the number of voxels to which it was connected. The measure of connectivity (degree, *D*) between a given voxel (*i*) and all other voxels (*j*) is given as the number of adjacent links (*d*_ij_), using the following equation:

(1) $$D_{i}=\sum^{\text{​}}d_{ij}\text{ Where j}=1,\;2,\;\ldots ,\text{ N},\text{ i }\neq \text{j}$$

The voxels with higher *DC* values usually indicate their central roles in the functional integrity of the whole-brain networks, referred to as hubs in terms of graph theory; (4) The *DC* value of each voxel was then converted to *Z* scores with Fisher’s *Z*-transformation for standardization purposes. Finally, all individual *DC* maps were spatially smoothed with a Gaussian kernel (full-width at half-maximum (*FWHM* = 6 mm).

### 4.5. ALFF Calculation

The *ALFF* was calculated using REST software. Briefly, the time series was transformed to frequency domains using fast Fourier transforms, and the power spectrum was obtained. Because the power of a given frequency is proportional to the square of the amplitude of the frequency component, the power spectrum was square-rooted and then averaged across 0.01–0.08 Hz at each voxel. This averaged square root was taken as the *ALFF*. For standardization purposes, the *ALFF* of each voxel was divided by the global mean *ALFF* value. Finally, all subject-level *ALFF* maps were spatially smoothed with a Gaussian kernel (*FWHM* = 6 mm).

### 4.6. Statistical Analysis

#### 4.6.1. Comparison of Demographic and Clinical Data and Head-Motion Parameters

Student’s *t*-test and analysis of variance (ANOVA), as appropriate, were used to compare the demographic and clinical data and head-motion parameters between the subgroups. The software package utilized for this analysis was Statistical Package for Social Sciences for Windows 16.0 (SPSS Inc., Chicago, IL, USA). *p* < 0.05 was taken to be indicative of statistical significance.

#### 4.6.2. Voxel-Wise Comparison of *DC* Maps and *ALFF* Maps

For comparisons of *DC* maps, a two-factor ANCOVA model was specified using SPM8 [65], with sex (male, female) and diagnosis (FES, HC) as the between-subject factors, and age as covariates. The sex by diagnosis interaction, and the main effects of sex and diagnosis were tested. When interaction effects occurred, *post hoc* pair-wise comparisons were performed using two-sample *t*-tests within the interaction masks. The regions showing selective *DC* alterations in either male (*i.e.*, in HCm *vs.* FESm or HCf *vs.* FESf comparisons) in the *post hoc* comparisons were defined as seeds for further network analyses. To illustrate the topography of the candidate hubs, we also conducted a series of one-sample *t*-tests for the *DC* maps of each group, with age and years of education as covariates.

Comparisons of the *ALFF* maps were as described above for the *DC* maps, except that one-sample *t*-tests were not used.

For all voxel-wise comparisons, corrections for multiple comparisons were made by Monte Carlo simulations using the AlphaSim program in the REST software. A corrected significance level of *p* < 0.05 was obtained with a combined *p* < 0.001 and cluster size >351 mm^3^ (13 voxels). To further evaluate the reproducibility of our findings, we also assessed the stability of our main results (the sex by diagnosis interaction) by applying *p* values of 0.005 and 0.01 (see Figures S3 and S4).

### 4.7. Correlation Analysis

For all regions showing sex-specific functional alteration or a main effect of diagnosis, the correlations of the mean *DC* (or *ALFF*) values in these regions with the symptoms (PANSS total and subscale scores) and with the age of illness onset were determined to investigate the clinical correlates of the functional abnormality patterns. For ROIs (Region of interest) with sex specific functional alterations (*i.e.*, Put_R, MFG and vmPFC), correlations were calculated in male and female patient group respectively and also in all FES patients (combining male and female). For the ROIs with main effect of diagnosis (bilateral putamen, IPL, PCC and MTG), correlations were calculated in all FES patients. The threshold was set at 0.006 (0.05/8), corrected for multiple comparisons using the Bonferroni correction for the number of ROIs (*i.e.*, 8 ROIs in this study) [66].

### 4.8. Network Analyses

To reconstruct the networks associated with sex-specific *DC* alterations a seed-based functional connectivity analysis was conducted. The regions showing sex-specific *DC* alterations were selected as seeds [25], and the functional connectivity map (FCmap) for each seed was constructed in HCs and thresholded with *p* < 0.05 (AlphaSim corrected, *p* < 0.001 and cluster ≥ 13). These FCmaps were considered as the network linked to the seeds.

## 5. Conclusions

In conclusion, the present study revealed a sex-specific pattern of functional aberration in patients with FES, and these sexually specific aberration patterns were associated with the clinical features in male and female patients.

## Supplementary Materials

Supplementary materials can be found at http://www.mdpi.com/1422-0067/16/07/16125/s1.
