# Supplementary material for: Sex-Specific Patterns of Aberrant Brain Function in First-Episode Treatment-Naive Patients with Schizophrenia
Source: Int J Mol Sci. 2015 Jul 16;16(7):16125–43. doi: 10.3390/ijms160716125 (PMC4519942; doi:10.3390/ijms160716125)
Supplement: Supplementary file 1 [file ijms-16-16125-s001.pdf]

Supplementary Information

1. The Influence of Short Period Antipsychotic Treatment

Twenty-eight of the FES patients (22.58%; 15 male and 13 female) in this study had been minimally treated with antipsychotic medication prior to MRI scanning at low dosage (ranging from 25–75 mg of chlorpromazine daily dose equivalents) for a duration of <3 days. These patients were included to maintain statistical power to demonstrate group differences. It is not clear whether antipsychotic treatment, thought with minimal dosage and short period, may substantially change the intrinsic neural activity of patients. To evaluation the potential effects of minimal treatment, we rerun the statistical analysis using only the DC/ALFF maps of neuroleptic-naive FES patients (46 male, 50 female), the threshold were set at $p < 0.001$. The sex by diagnosis interaction maps are shown in Figure S1. The interaction maps remains unchanged after excluded the patients with minimal treatment, suggest that minimal treatment did not qualitatively affect our results.

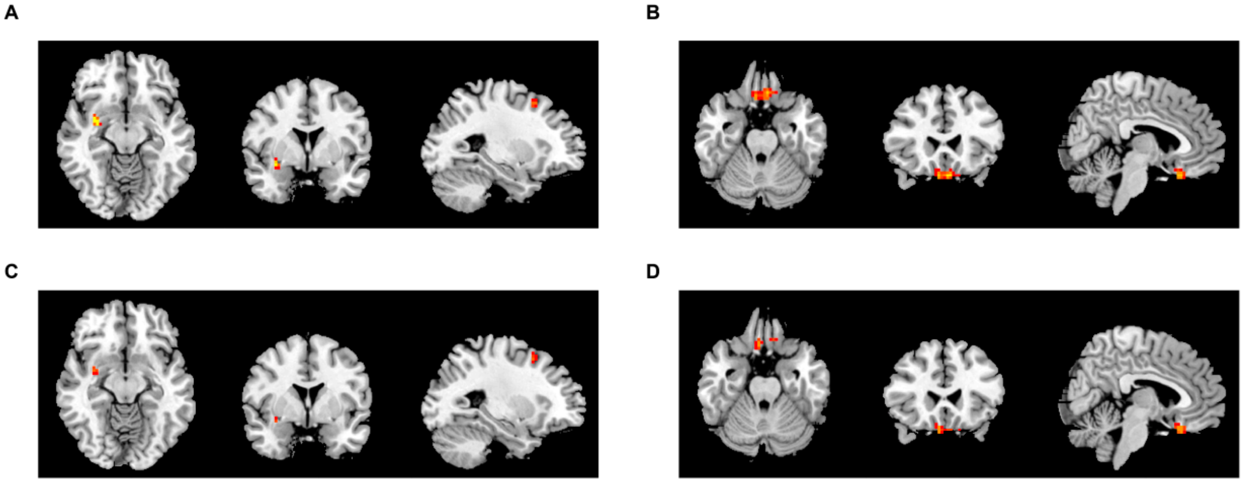

Figure S1. The sex by diagnosis interaction maps while included or excluded the minimal treated FES patients. (**A**) Interaction on DC with treated patients included; (**B**) Interaction on ALFF with treated patients included; (**C**) Interaction on DC with treated patients excluded; (**D**) Interaction on ALFF with treated patients excluded.

2. The Influence of Different Threshold in Computing the Degree Centrality

We selected $r > 0.25$ so as to employ a relatively conservative threshold and thereby minimize the contributions of spurious correlations. This high threshold may disadvantage regions with many weak (but true) correlations. To determine whether our main results depend on these choices, we recomputed the maps for correlation thresholds $r > 0.00$, $r > 0.10$, $r > 0.15$, $r > 0.20$, $r > 0.25$, and $r > 0.30$ for our participants, and rerun the statistics. The sex by diagnosis interaction maps for different r thresholds are shown in Figure S2.

The topography of the interaction maps was qualitatively similar across all r thresholds, with the interaction effects being more robust for the more stringent correlation thresholds.

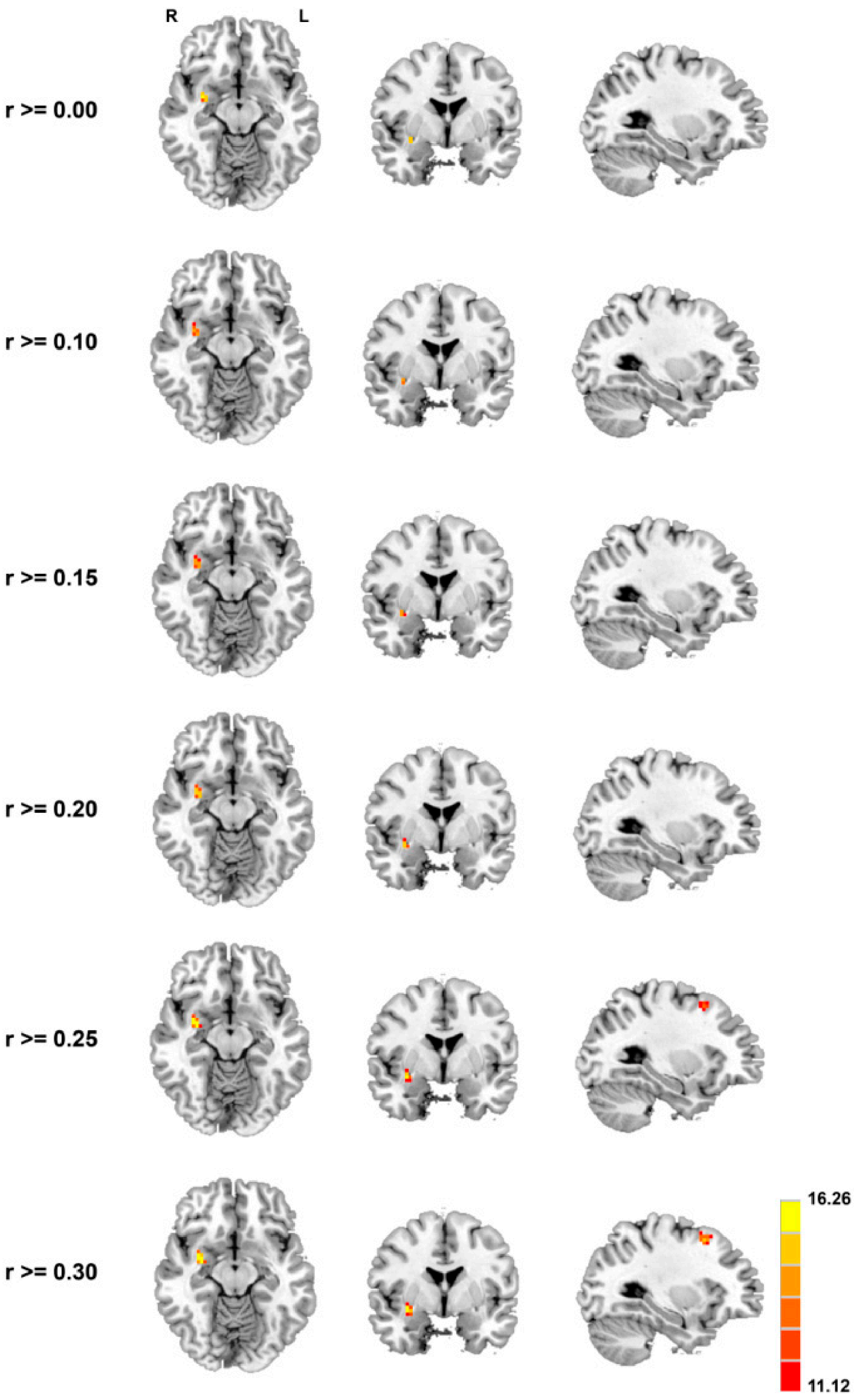

Figure S2. The sex by diagnosis interaction maps of DC for different correlation thresholds (From top to bottom panel, the cut off r = 0.00, 0.10, 0.15, 0.20, 0.25 and 0.30 separately).

3. The Reproducibility of Our Main Results

To further evaluate the reproducibility of our results, we assessed the stability of our main results by applying different voxel level p values of 0.001, 0.005 and 0.01(see Figures S3 and S4). The topological architecture of our main results was highly similar.

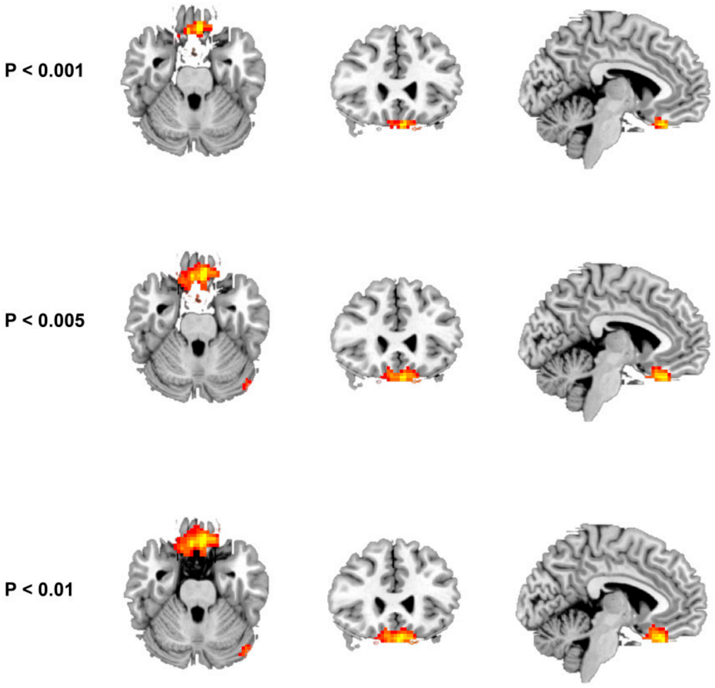

Figure S3. The sex by diagnosis interaction of ALFF maps at different p thresholds.

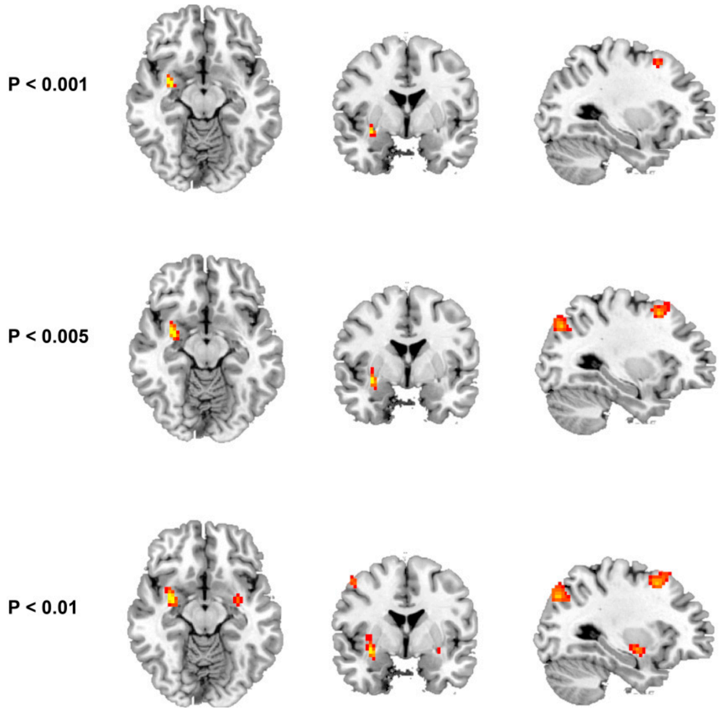

Figure S4. The sex by diagnosis interaction of DC maps at different p thresholds.

4. The Maps of the "MFG-related Network" and "Put_R-related Network" in Four Groups of This Study

The networks connected to MFG and Put_R were in qualitatively similar in all 4 groups of this study, as showed in Figures S5 and S6.

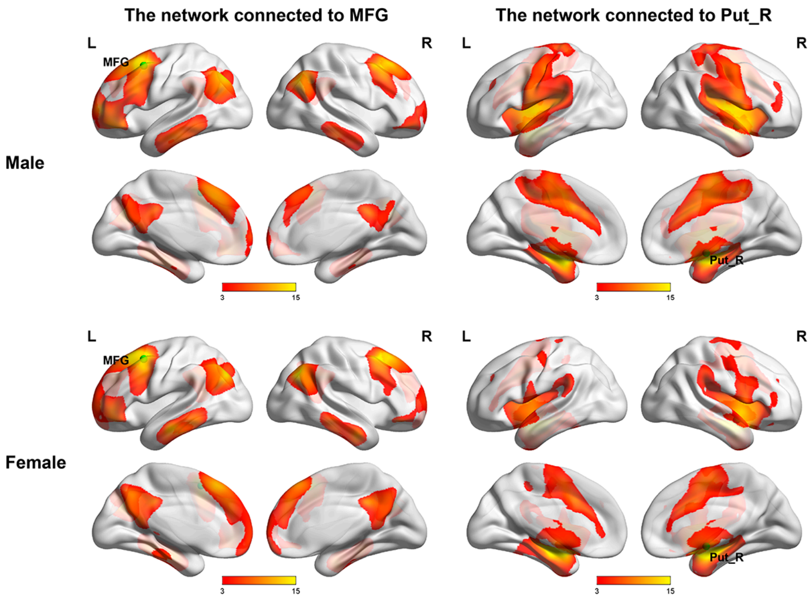

Figure S5. Functional networks that connected to MFG (**left panel**) and Put_R (**right panel**) in male (**top panel**) and female (**bottom panel**) healthy controls. The seed regions showed as green dots. The color bars represent the *t*-values of voxel-wise one-sample *t*-tests. L, left hemisphere; R, right left hemisphere; Put_R, right putamen; MFG, middle frontal gyrus.

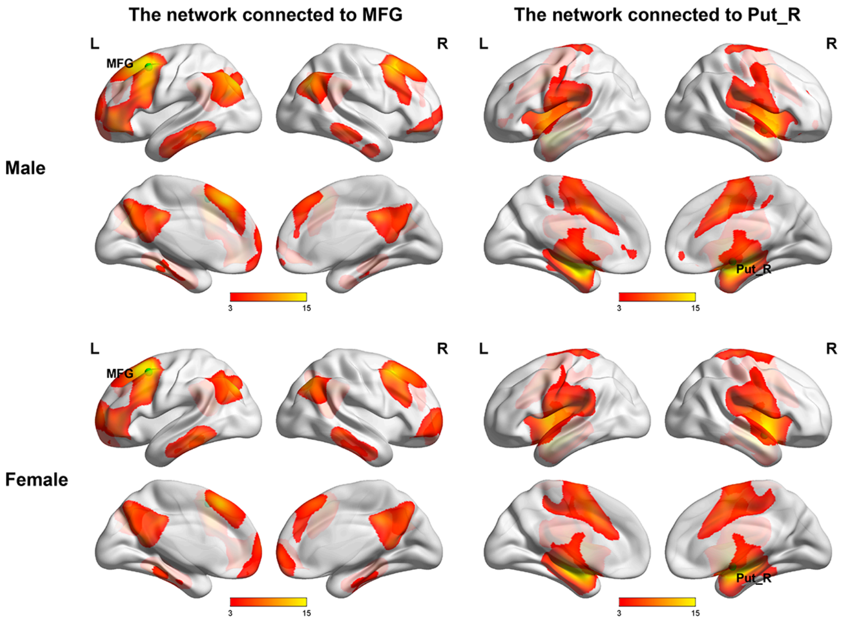

Figure S6. Functional networks that connected to MFG (**left panel**) and Put_R (**right panel**) in male (**top panel**) and female (**bottom panel**) FES patients. The seed regions showed as green dots. The color bars represent the *t*-values of voxel-wise one-sample *t*-tests. L, left hemisphere; R, right left hemisphere; Put_R, right putamen; MFG, middle frontal gyrus.

5. The Superimposed Maps of the "MFG-related Network" and "Put_R-related Network" With Canonical SMN and DMN

The distribution of MFG-related network involved regions mostly overlapped with the default mode network, and the Put_R-related network was similar to the previously reported sensorimotor network. To parcellate the distribution of these two networks we get from the network analyses, we overlapped these two network with template of DMN and SMN (motor network). Details about how these templates were made can be found here (http://www.brainnexus.com/resources/resting-state-fmri-templates).

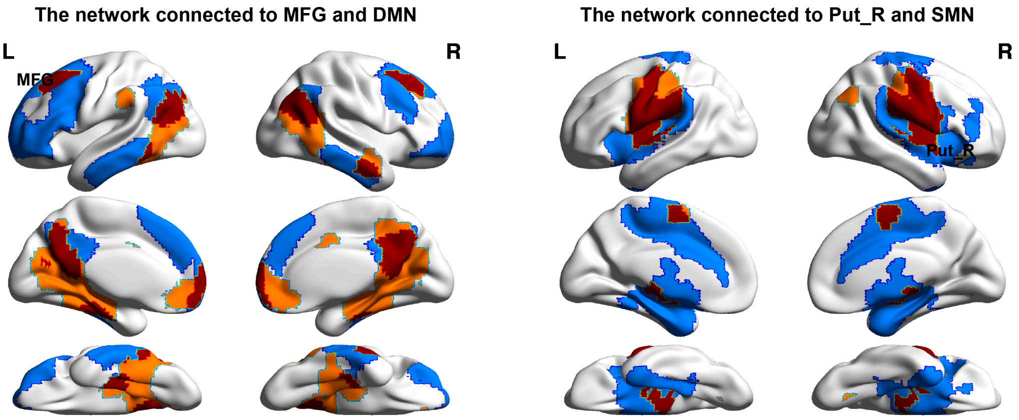

Figure S7. The overlap view of MFG-related network (blue) with a prior template of default mode network (DMN, orange) (the **left panel**); and Put_R-related network (blue) with a template of sensorimotor network (DMN, orange) (the **right panel**). The overlap regions were shown in caramel.
